# Fatty Acid Profile as an Indicator of Larval Host for Adult *Drosophila suzukii*

**DOI:** 10.3390/insects11110752

**Published:** 2020-11-03

**Authors:** Nik G. Wiman, Heather Andrews, Erica Rudolph, Jana Lee, Man-Yeon Choi

**Affiliations:** 1 North Willamette Research and Extension Center, Oregon State University, 15210 NE Miley Rd, Aurora, OR 97002, USA; heather.andrews@oregonstate.edu (H.A.); rudolphe@oregonstate.edu (E.R.); 2 Horticultural Crops Research Unit, USDA-ARS, 3420 NW Orchard Ave, Corvallis, OR 97330, USA; jana.lee@ars.usda.gov (J.L.); mychoi@ars.usda.gov (M.-Y.C.)

**Keywords:** dietary routing, random forests, invasive insect pest, spotted-wing drosophila

## Abstract

**Simple Summary:**

Spotted-wing drosophila, *Drosophila suzukii*, is an invasive pest of soft-skinned fruits. Adult female flies oviposit, or lay eggs, into fruits where the larvae develop, making infested fruit unmarketable. The flies rely on alternative hosts, both cultivated and wild, to survive and maintain populations throughout the year. Better understanding of how the flies migrate between different hosts could be beneficial to improving management of the pest in crops. This study demonstrates potential to discriminate larval host of adult flies by analysis of fatty acids carried from the larvae to the adult stage in the body using a machine learning algorithm as an alternative to linear discriminant methods. Our study shows that fatty acids in adult flies can be used to determine larval host and that the machine learning algorithm can perform the discriminant analysis without making any assumptions about the data.

**Abstract:**

*Drosophila suzukii* is a severe economic invasive pest of soft-skinned fruit crops. Management typically requires killing gravid adult female flies with insecticides to prevent damage resulting from oviposition and larval development. Fruits from cultivated and uncultivated host plants are used by the flies for reproduction at different times of the year, and knowledge of *D. suzukii* seasonal host plant use and movement patterns could be better exploited to protect vulnerable crops. Rearing and various marking methodologies for tracking movement patterns of *D. suzukii* across different landscapes have been used to better understand host use and movement of the pest. In this study, we report on potential to determine larval host for adult *D. suzukii* using their fatty acid profile or signature, and to use larval diet as an internal marker for adult flies in release-recapture experiments. Fatty acids can pass efficiently through trophic levels unmodified, and insects are constrained in the ability to synthesize fatty acids and may acquire them through diet. In many holometabolous insects, lipids acquired in the larval stage carry over to the adult stage. We tested the ability of a machine learning algorithm to discriminate adult *D. suzukii* reared from susceptible small fruit crops (blueberry, strawberry, blackberry and raspberry) and laboratory diet based on the fatty acid profile of adult flies. We found that fatty acid components in adult flies were significantly different when flies were reared on different hosts, and the machine learning algorithm was highly successful in correctly classifying flies according to their larval host based on fatty acid profile.

## 1. Introduction

*Drosophila suzukii* (Matsumura) (Diptera: Drosophilidae) is a severe economic invasive pest of soft-skinned fruit crops over its introduced range, which includes temperate horticultural crop production regions in North America, Europe and South America [1]. This invasive pest is adapted to attack ripening fruit unlike most drosophilids, which are restricted to attacking ripe, overripe, or damaged fruit [2]. Management of the pest in crops has largely relied on killing gravid adult female flies with insecticides to prevent damage resulting from oviposition and larval development [3,4]. Complicating the management effort are population dynamics, phenology, and life history of the pest. Reproductive capacity for *D. suzukii* is high, generations are rapid, and a complex of cultivated and uncultivated host plants are used by the flies for reproduction at different times of the year [5,6,7,8,9,10,11]. Adult flies are capable of long-distance migrations, giving them access to a wide range of environments and host plants [12]. Phenotypic plasticity in *D. suzukii* allows greater environmental adaptation and improves winter survival [13,14,15].

Management of *D. suzukii* has focused on protecting ripening crops at the point where they become vulnerable to the pest, but knowledge of seasonal host plant use and movement patterns could be better exploited to protect vulnerable crops. In northern latitudes, several studies have suggested that population densities are at their annual minimum in early spring [16,17], suggesting that one key to management could be understanding the origins of the initial population that immigrates into managed crops. This understanding may provide an opportunity to manage the pest efficiently before the populations increase, or to better recognize spatial invasion patterns into crops for targeted monitoring and management. Other studies of movement of the pest in the environment could be valuable for determining attraction to volatiles, evaluation of biological control, and other research questions.

Methodologies for tracking movement patterns of *D. suzukii* across different scales have used a host of marking techniques. These include use of immunomarking techniques where flies self-mark by coming into contact with a host plant treated with a protein [12,18,19], mark–release–recapture experiments with immunomarkers where protein is applied to flies prior to release [20], marking flies with fluorescent powder [21], and gut content analysis [22,23]. Each of these methods can inform about the spatial dynamics of the pest, but each method also has limitations. 

In this study, we report on the potential to determine the larval host of adult *D. suzukii* using fatty acid profiles or signatures, and potential use of fatty acid profiles as an internal marker in adult flies. Fatty acids are carboxylic acid molecules with long aliphatic hydrocarbons containing 4–28 C atoms and 0–3 C=C double bonds in the chain [24]. In insects, free fatty acids are needed as precursors to the signaling molecules eicosanoids, for pheromone synthesis, as well as for cuticular waxes and phospholipids [25]. They are abundant in the insect fat body in the form of triglycerides, the major source of stored energy, and as cholesteryl esters, which serve other important physiological roles [26]. Fatty acids can pass efficiently through trophic levels unmodified in a process termed dietary routing [27]. Insects are particularly constrained in their ability to synthesize fatty acids, and with exceptions cannot generally synthesize polyunsaturated fatty acids de novo [28]. Dietary routing and abundance in stored fat make fatty acids useful as biomarkers of trophic transfer in food web ecology, a technique that has been embraced far more in the realm of aquatic compared to terrestrial ecosystems [29]. 

Because fatty acid content in insects is determined by diet, fatty acid signatures can be useful as biomarkers to determine other aspects of insect ecology than trophic transfer. For phytophagous pests, the fatty acid profile represents host plant use, and for crop pests this technique can be exploited to improve knowledge of pest movement in the landscape. For example, navel orangeworm moths (*Amyelois transitella* (Walker) (Lepidoptera: Pyralidae)) have distinct fatty acid signatures depending on the larval host crop, and fatty acid profile analysis allowed researchers to better understand source-sink dynamics of the pest between susceptible nut crops [30]. Another way that fatty acid profiles could be used in pest management is as a simple marker for differentiating laboratory reared insects released in the field from their wild counterparts, which would be useful for mark–release–recapture and other experiments. This would require that laboratory diets be manipulated to impart distinctive fatty acid profiles to distinguish released insects from their wild counterparts.

In this research, we tested the potential to discriminate larval hosts of *D. suzukii* by analyzing fatty acid profiles of adult flies. A major question for the application of the fatty acid profiles to a pest such as *D. suzukii* is the observation that some of the different fruits used by the pest are quite similar, and may not result in distinctive fatty acid profiles in the adults. We wanted to determine whether there is potential to ultimately use the method on adult flies captured from the field to determine where their larvae developed. Particularly at critical time points during the growing season, the ability to determine the host plant of origin for the flies found attacking crops could provide useful information that could improve pest management. A secondary objective was to determine if the diets used to rear flies in the lab could be useful as a simple marking technique for mark–release–recapture experiments where the mark is acquired through the larval diet. Discriminant analysis of fatty acid profiles in adult flies is the logical statistical analysis for determining larval host based on levels of individual fatty acids in adult flies. Rather than trying to conform data to assumptions of traditional linear discriminant methods, we used a machine learning algorithm to discriminate larval hosts of adult *D. suzukii* by the fatty acid profile of each fly.

## 2. Materials and Methods

### 2.1. Insect Rearing

Laboratory colonies of *D. suzukii* were reared on artificial diet in the laboratory at the Oregon State University North Willamette Research and Extension Center in Aurora, OR (16:8 L:D, 20 °C) in BugDorm Cages (MegaView Science Co., Taichung, Taiwan). The diet consisted of 45 g agar, 125 g cornmeal, 200 g sucrose, 70 g nutritional yeast, 4.7 L dH_2_O, 17.7 mL proprionic acid, 3.3 g methylparaben, 33.3 mL ethanol (95%) with sprinkles of baker’s yeast on the surface [31]. Colonies originated from field-collected flies in the northern Willamette Valley, Oregon USA, a major production region for small fruits such as blueberry, blackberry and raspberry, and were regularly supplemented with wild flies to avoid inbreeding. 

Ripe fruits susceptible to *D. suzukii* were obtained from research plots at the North Willamette Research and Extension Center, Aurora, OR, including fresh strawberry (*Fragaria* × *ananassa* (Weston)), blueberry (*Vaccinium corymbosum* L.), raspberry (*Rubus idaeus* L.) and blackberry (*Rubus fruticosus* L.) in 2018 and 2019. If unavailable from the field, fruit was sometimes purchased. Raspberries, blackberries, blueberries, strawberries were rinsed, patted dry, and then placed into individual 946 mL (2 pt) plastic containers that were lined with a damp paper towel to maintain moisture. Approximately 30 male and female *D. suzukii* were aspirated from the lab colony, placed into the containers with approximately 80 g of fruit or artificial diet, with a mesh cover. Flies were allowed approximately 72 h to oviposit into fruit, and were then removed from containers along with paper towels to prevent larvae from feeding on juice soaked towels and thus potentially altering the lipid makeup. Cotton dental wicks were added to raspberry containers to absorb excess juice. Upon emergence from the provided larval diet, adults were aspirated, then placed in individual microcentrifuge tubes and frozen at −80 °C at the USDA-ARS Horticultural Crops Unit in Corvallis, OR for fatty acid profile analysis.

### 2.2. Fatty Acid Analysis

Flies in microcentrifuge tubes were removed from the freezer and allowed to thaw. For total lipid extraction 50 µL of 2% sodium sulfate was added to each 1.5 mL centrifuge tube, and a plastic pestle was used to crush each fly. Pestles were rinsed into tubes with 450 µL chloroform/methanol (2:1). After centrifugation for 3 min at 13,000 rpm, the resulting extract (approximately 480 µL) was prepared by base methanolysis. The preparation of fatty acid methyl esters (FAMEs) from the lipids extracted was modified from the previous studies [32,33]. One-hundred mg of pentadecanoic acid as an internal standard was added to each sample. After the solvent dried, 400 µL of 1 M KOH (in 70% ethanol) was added to the vial, which was then incubated at 90 °C for 1 h. After cooling, 400 µL 1M HCl was added to samples, which were vortexed vigorously, then lipid was extracted with 600 µL hexane. The upper phase containing lipid was transferred to a new clean glass vial. After hexane evaporated, 200 µL of boron trifluoride diethyl etherate (in 10% methanol) was added, and samples were incubated 20 min at 37 °C. Purified water (200 µL) was then added to sample vials, vortexed, then 200 µL hexane was added. After being vortexed again, the upper phase was collected for gas chromatography (GC) or GC/Mass spectroscopy (MS) analysis. FAMEs were then analyzed using a GC-MS (Agilent 7890B GC system) coupled with a Mass Selective Detector (Agilent 5977B) (Santa Clara, CA, USA). The GC-MS was equipped with a capillary column (HP-5, 30 m × 0.25 mm; Agilent). The oven was temperature programmed to 80 °C for 1 min, then to increase by 5 °C/min to 300 °C and held for 10 min. FAMEs were analyzed in the scan ion monitoring mode using an NIST 2017 MS Library (Agilent Technologies). Once identified, quantity of FAMEs was determined by comparison of the internal standard using the GC (Agilent 7890B) equipped with a flame ionization detector and a HP-5 capillary column (30 m × 0.25 mm; Agilent, Santa Clara, CA, USA). The oven temperature followed the same program described above.

### 2.3. Statistical Analysis

All statistical analyses and graphical outputs were produced in R [34]. Each fatty acid component was calculated as a proportion of the total fatty acid extracted from each fly, and therefore the proportional response variables were not considered to represent normally distributed variables. Therefore, we used generalized or nonparametric data analyses for all analyses. To test differences in the mean proportion of each fatty acid of flies reared on the different diets, we used generalized linear models (GLM) with a quasibinomial family and logit link function. To test significance of GLM models, we performed analysis of deviance to produce a p-value for the asymptotic chi-square test statistic based on the deviance. Following a significant GLM, we used Tukey multiple comparisons to compare mean proportions using R package ‘glht’ [35]. To visualize potential groupings of flies by host, nonmetric multidimensional scaling (NMDS) ordination using the Bray–Curtis dissimilarity index was constructed based on fatty acid proportions for flies from different hosts using R package ‘vegan’ [36]. Finally, to analyze the potential to determine larval host of adult flies of unknown origin, we used the random forest machine learning algorithm using the R package ‘randomForest’ [37]. This package executes the random forest algorithm of Breinman [38] based on original code adapted to R from Fortran. The number of variables randomly sampled at each split in random forest was determined from the value of m_try_ that minimized the Out-of-Bag (OOB) error estimate. A stratified random sample representing 60% of the data for each larval host was selected for training, and the remaining 40% of data were used to test the ability of the model to discriminate the larval host for each fly in the data set. We used 200 trees in the algorithm. Raw fatty acid profile data from the project can be found in the supplemental file (Table S1).

## 3. Results

We analyzed fatty acid profiles of individual *D. suzukii* flies reared from blueberry (n = 34), blackberry (n = 49), raspberry (n = 10), strawberry (n = 48) and artificial diet (n = 49). Sex ratio of analyzed flies was 0.84:1.0 m:f. Six fatty acids were detected from adult *D. suzukii*: tetradecanoic acid (14:Acid), 9,16-hexadecanoic acid (9–16:Acid), hexadecanoic acid (16:Acid), 9,12-octadecadienoic acid (9,12–18:Acid), 9,12,15-octadecatrienoic acid (9,12,15–18:Acid), and octadecanoic acid (18:Acid). All GLMs for the proportion of total FAME represented by each individual fatty acid for the different larval diets were significantly different from the null model in analysis of deviance, indicating there were differences in the proportion of fatty acid in flies reared from each diet (Table 1).

**Figure 1:**
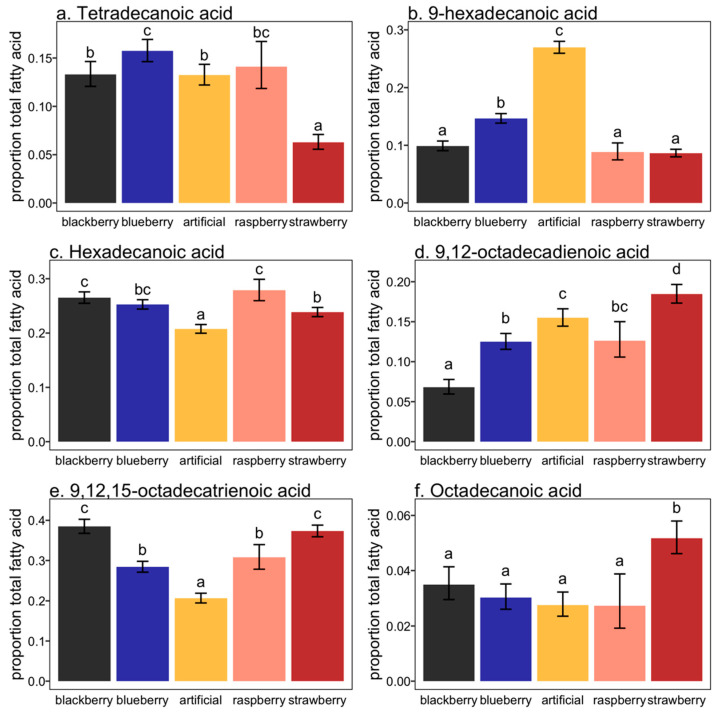
Mean proportion (±SEM) of each fatty acid component for *Drosophila suzukii* adults reared from different fruit hosts and artificial diet. Different letters indicate significant differences (Tukey HSD; *p* < 0.05).

While fatty acid models all differed from the null models, fatty acids levels were not necessarily significantly different for each larval diet within each model. A larval diet of blueberry or raspberry resulted in the highest levels of 14:Acid in the adult flies, though the raspberry diet did not result in statistically more or less 14:Acid acid than blackberry or artificial larval diets. The strawberry larval diet resulted in the lowest level of 14:Acid acid in adults (Figure 1a). Proportion of total fatty acids attributed to 9–16:Acid was consistent for flies that were reared from the blackberry, raspberry and strawberry, but was elevated for blueberry and the highest level was in flies reared from artificial diet (Figure 1b). A relatively high proportion of total fatty acids was represented by 16:Acid in flies reared from all the diets, though the flies reared from blackberry and raspberry had the highest levels, and flies reared from artificial diet had the lowest levels (Figure 1c). Flies reared from strawberry had the highest proportion of fatty acids attributed to 9,12–18:Acid, while flies reared from blackberry had the lowest levels (Figure 1d). An unsaturated 18-carbon fatty acid, 9,12,15–18:Acid was another major component of total fatty acids in most diets, although flies reared from the artificial diet had significantly less of this component than the berry diets (Figure 1e). Blackberry and strawberry diets had the highest levels of 9,12,15–18:Acid, while blueberry and raspberry had intermediate levels. Finally, 18:Acid represented a very minor proportion of the total fatty acids, and was present at highly consistent levels between most berry diets, except that flies reared from strawberry had significantly higher levels (Figure 1f). 

**Figure 2:**
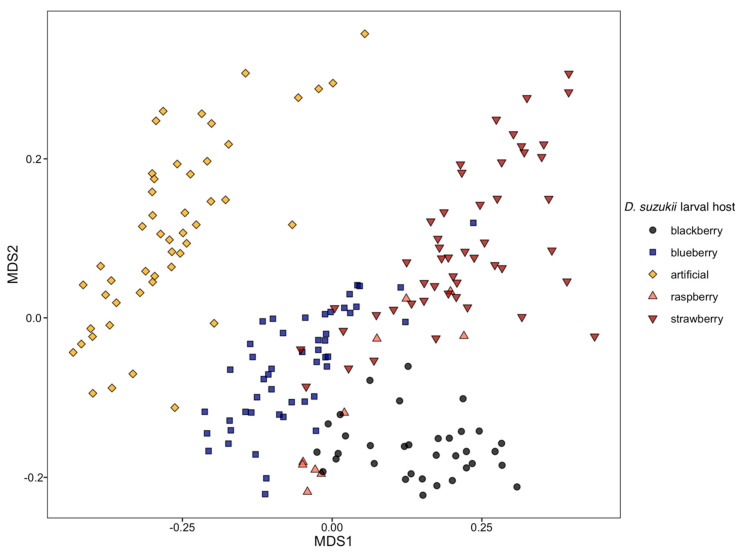
NMDS (2-dimensional) plot for proportion of fatty acids of *Drosophila suzukii* adults reared from different larval diets (Bray–Curtis dissimilarity index).

For the NMDS analysis, the best solution was found after 20 runs, with a stress value of 0.106. Flies reared from artificial diet formed a tight group with no overlap with other points (Figure 2). Flies reared from blackberry were tightly clustered and overlapped only with flies reared from raspberry; there were no overlapping points from flies reared from blueberry and blackberry hosts and blackberry and strawberry hosts. Flies from other berry hosts were less distinctly grouped, particularly flies reared from raspberry, which did not overlap with flies from blueberry, but flies reared from raspberry were not well separated from flies reared from either blackberry or strawberry.

**Figure 3:**
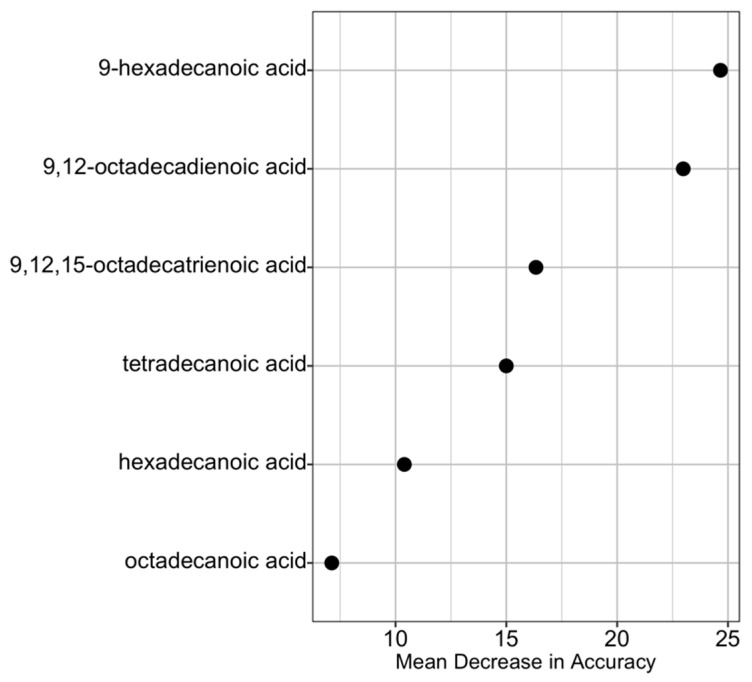
Ranked importance of the fatty acids in the random forests model using Gini Index.

In optimizing the random forest analysis, the value for m_try_ resulting in the lowest OOB (8.9%) was 2, which was input into the random forest classification algorithm. There were 113 rows of data representing individual flies reared from different larval hosts in the training data set and 78 rows in the test data set. Sum reduction in Gini impurity suggested that the two most important predictor variables for the random forest model were 9-hexadecanoic acid and 9,12-octadecadienoic acid (Figure 3), both fatty acids showed some of the highest heterogeneity in mean proportion of total FAME from different hosts (Figure 1b,d).

Applying the random forest model to the smaller (40% of the complete data set) testing data set resulted in OOB error rate of 7.33%, with good classification of flies from different hosts (Table 2). From the 78 flies in the test classification data set, 67 (86%) were correctly classified by random forests. The random forests algorithm correctly identified 93% of flies reared from blackberry, with 7% misclassified as raspberry. Flies reared from blueberry were also classified with high accuracy (95%), with 5% of flies misclassified as originating from strawberry. One fly of 20 reared from blueberry was misclassified as having been reared from artificial diet, and 1 of 20 flies reared from artificial diet were misclassified as having been reared from strawberry. Flies reared from strawberry were most difficult to classify, and the algorithm miscalculated 3 of 20 flies (15%) reared from strawberry as having been reared from blueberry.

## 4. Discussion

Hydrocarbon profiles including cuticular hydrocarbons and internal fatty acids play important physiological roles in insects and are increasingly used as chemical indicators of morphological, physiological, behavioral and ecological attributes [39,40,41,42,43,44]. This study demonstrated dietary routing of fatty acids from fruits of host plants consumed by larval *Drosophila suzukii* into adult flies. Results suggest analysis of fatty acid profiles in adult *D. suzukii* is a potential methodology to determine movement patterns and host use behavior by identifying the larval host of field-collected flies. This could improve our understanding of landscape and phenological population dynamics of this pest. One limitation of the technique is that there must first be training data to represent fatty acid profiles for flies reared from the potential host plants in order to classify insects collected from the field in traps or other collection methods. It is time consuming, but not difficult to collect host plant materials from wild or cultivated host plants to rear the pest from the fruits or expose them to gravid female *D. suzukii* in the lab. Fatty acid profile analysis performed on emerging adults can be used to build a library of profiles as a training data set. Multiple techniques have been established for collecting adult flies from the field, including use of commercial lures and traps allowing adults to be easily collected from an area of interest such as a berry field. The fatty acid profile of field-collected adult flies could then be classified using a discriminant analysis with the reference fatty acid profile library. Classification of field-collected adults based on a reference library of fatty acid profiles representing potential host plants would allow several interesting questions to be pursued. A rudimentary question for pest managers of crops susceptible to *D. suzukii* is to determine the source of flies attacking the crop, potentially facilitating the identification of important hosts contributing to pest problems in the crop.

Another implication of these results is that flies reared in the laboratory from different diets can be marked by fatty acid profiles for mark–release–recapture experiments. This eliminates some of the concerns of external marking techniques that can interfere with behavior in mark–release–recapture experiments. The artificial diets are particularly useful in their ease of use for rearing large numbers of flies in the laboratory, but different fruit diets could also be used to mark individuals for field release. While the artificial diet used in this study has been used in many studies, there are many variations in composition of artificial diets used to rear *D. suzukii* [45]. There may be opportunity to alter balance of fatty acids in artificial diets in order to manipulate fatty acid profile in adult flies to create multiple fatty acid profiles to serve as marks, or further opportunity to increase differentiation of fatty acid profiles of flies reared from artificial compared to fruit diets.

This research also demonstrated the use of the powerful machine learning algorithm random forests to classify *D. suzukii* adults according to fatty acid profile. The great advantage of this analysis is that it makes virtually no assumptions about data structure, and therefore provides a great alternative to linear classification methods. Indeed, to successfully use linear classification methods on fatty acid profile data where each fatty acid is represented as a proportion of total fatty acids, then data transformations must be used to attempt to meet assumptions of linear classification procedures such as linear discriminant analysis (LDA) [30]. As ubiquity of powerful machine learning and other alternatives to classical linear statistical methods increase, there should be less reliance on data transformations and efforts to make data conform to assumptions of traditional parametric statistical methods. However, one downside of embracing modern data science is the lack of familiarity of the scientific community in using and reviewing use of these methods.

Potential limitations of the dietary routing of fatty acids that were not explored in this research will be important for future applications to this and similar species. We did not examine the integrity of the fatty acid profile over time in adult *D. suzukii*, but we focused on relatively young flies. Potentially, the ability to discriminate adult flies to natal host could change over time as flies age and fatty acids are consumed, potentially at different rates, by metabolic processes. In *D. melanogaster*, the larval fat body persists through metamorphosis into the adult stage in the form of disassociated dispersed fat cells [46]. The larval fat cells eventually die in the adult and are replaced by the adult fat body. As older adult *D. suzukii* replace remnant larval fat cells, it is unclear if the fatty acid composition of the adult would continue to reflect the larval host, or whether the fatty acid composition of the adult fat body would be a confounding factor. While floral resources are used by adult *D. suzukii* for survival, these increased carbohydrate content and not lipids of adult flies [47]. However, nectar can include minute levels of amino and fatty acids [48] and may influence fatty acid synthesis in adults. Besides carbohydrate, adult *D. suzukii* are attracted to yeasts for feeding, and the acquisition of yeasts can enhance reproductive capacity of flies [49]. Yeasts can be extremely rich in lipids, particularly oleaginous yeasts, which have 20% or more of their biomass comprised of lipid. Furthermore, fatty acid profiles of yeasts can depend on the environment under which they develop [50]. Thus, yeast consumption by adults may be a major source for the adult fat body that could influence the ability to classify larval host by fatty acid profile. Finally, larval *D. suzukii* have been associated with multiple yeast species that influence larval survival [51]. These yeasts colonize fruit consumed by larvae and composition of yeasts in the larval diet may also affect the fatty acid profile in the adult insect. So even within fruits, yeast populations could have an influence on fatty acid profiles.

## 5. Conclusions

We evaluated the potential to measure dietary routing of fatty acids acquired during larval feeding in adult flies of the invasive pest *Drosophila suzukii* using fatty acid profiles. While further research is required, there is strong potential to use this technique to better understand population dynamics and behavior of *D. suzukii* to benefit management. The technique is likely to be particularly well-suited to insects that have single hosts for the larval stage and limited ability or opportunity to acquire fatty acids in the adult stage. While *D. suzukii* does not perfectly fit these parameters, the technique does show some promise. We also showed how the random forests machine learning protocol can be applied to classify samples without making assumptions about data or working with transformed data sets to attempt to meet assumptions of more conventional linear discriminant methods.

## Figures and Tables

**Table 1:** Analysis of deviance tables for generalized linear models for proportions of total fatty acids represented by individual fatty acids for *Drosophila suzukii* reared from different hosts.

Model	Source	df	Deviance	Residual df	Residual Dev	*p* Value
Tetradecanoic acid (14:Acid)	Null			190	5.034	
	Host	4	2.555	186	2.478	<0.0001
9-hexadecanoic acid (9-16:Acid)	Null			190	1.317	
	Host	4	7.563	186	8.880	<0.0001
Hexadecanoic acid (16:Acid)	Null			190	1.464	
	Host	4	0.529	186	0.935	<0.0001
9,12-octadecadienoic acid (9, 12-18:Acid)	Null			190	4.813	
	Host	4	2.691	186	2.122	<0.0001
9,12,15-octadecatrienoic acid (9,12,15-18:Acid)	Null			190	6.656	
	Host	4	4.534	186	2.123	<0.0001
Octadecanoic acid (18:Acid)	Null			190	1.872	
	Host	4	0.490	186	1.382	<0.0001

**Table 2:** Random forest model (number of trees: 200; no. variables tried at each split: 2; OOB error rate: 7.33%) performance classifying *Drosophila suzukii* adults according to larval diet in the test data set, indicating the number classified and the percentage correct or incorrect classification in parentheses.

	Predicted					
**Observed**	Blackberry	Blueberry	Artificial	Raspberry	Strawberry	Error
**Blackberry**	13 (93%)	0 (0%)	0 (0%)	1 (7%)	0 (0%)	1 (7%)
**Blueberry**	0 (0%)	19 (95%)	0 (0%)	0 (0%)	1 (5%)	1 (5%)
**Artificial**	0 (0%)	2 (10%)	16 (80%)	0 (0%)	2 (10%)	4 (20%)
**Raspberry**	0 (0%)	1 (25%)	0 (0%)	1 (25%)	2 (50%)	3 (75%)
**Strawberry**	0 (0%)	2 (10%)	0 (0%)	0 (0%)	18 (90%)	2 (10%)

## Supplementary Materials

The following are available online at https://www.mdpi.com/2075-4450/11/11/752/s1, Table S1: Raw data.
